# 2nd South Africa–United Kingdom cardiovascular workshop

**Published:** 2012-11

**Authors:** Roisin Kelly-Laubscher, Gideon Burger, Neil Davies, Anna-Mart Engelbrecht, Karen Sliwa, Sandrine Lecour, Hans Strijdom, Derek Hausenloy

**Affiliations:** Department of Human Biology, University of Cape Town, South Africa; Lasec, Cape Town, South Africa; Cardiovascular Research Unit, University of Cape Town, South Africa; Department of Physiological Sciences, University of Stellenbosch, South Africa; Hatter Institute for Cardiovascular Disease in Africa, University of Cape Town, South Africa; Hatter Institute for Cardiovascular Disease in Africa, University of Cape Town, South Africa; Division Medical Physiology, University of Stellenbosch, South Africa; Hatter Cardiovascular Institute, University College, London, United Kingdom

## Highlighting cardiovascular research in South Africa

It has been predicted that by the year 2030, the prevalence of death from cardiovascular disease (CVD) will have increased significantly.^1^ The perception that CVD is not relevant to developing countries needs to be redressed. In fact, South Africa (SA) is undergoing a more rapid increase in the prevalence of CVD than the developed countries, mainly due to urbanisation and the threatening increase in incidence of obesity and diabetes.^2^ Therefore there is a need for cutting-edge research with the potential to produce better therapies for the treatment of CVD in Africa.

To facilitate this cutting-edge research, it is imperative that we create a skills base capable of producing such research. The UK–SA workshops are events that we feel facilitate this process by establishing north–south collaboration, enticing promising young cardiovascular scientists to South Africa, and introducing our own students to a large network of scientists and opportunities for development.

The second UK–SA cardiovascular research workshop was held in Cape Town, South Africa in August 2012. The workshop was hosted by the South African Society for Cardiovascular Research (SASCAR), a special-interest group of the South African Heart Association, at the Chris Barnard building, University of Cape Town (UCT). The main organisers of this event were Prof Sandrine Lecour, head of the cardioprotection group at the Hatter Institute for Cardiovascular Research in Africa, UCT, and Dr Derek Hausenloy, British Heart Foundation senior clinical research fellow at the Hatter Institute, University College London.

The purpose of this joint UK–SA cardiovascular research workshop was to highlight the work of our young clinical and basic science researchers and to promote fruitful cardiovascular research collaborations between the UK and South Africa through the auspices of the European Society of Cardiology (ESC), the University of Cape Town and the SASCAR. The main research themes of the workshop represented the major overlapping research interests in the UK and South Africa, and included novel cardioprotective therapeutic approaches, cardiomyopathies, cardiovascular risk factors, clinical cardiovascular research, signalling pathways in cardioprotection and myocardial ischaemia–reperfusion injury. There were also presentations on problems more relevant locally, such as rheumatic heart disease, myocarditis in HIV-associated cardiomyopathies, and the effects of antiretroviral therapy on cardiac contractile function.

The workshop had over 65 delegates, including invited faculties from Europe, the USA and South Africa, five invited and sponsored cardiovascular research PhD students from academic institutions in Europe, and approximately 50 South African cardiovascular researchers and students. At the beginning of the week, the five invited European students had the opportunity to visit and spend time in research laboratories in South Africa. The workshop took place on a Thursday and Friday and each of the European and South African students had the opportunity to present their work.

The SA–UK workshop targets clinical and basic science PhD students in their later years of study. This is a critical time when students are trying to decide what they will do after completing their PhD. Unfortunately some very good scientists leave cardiovascular research after obtaining a PhD because they do not see any opportunities available to them. The SA–UK workshop introduces them to a network of potential national and international postdoctoral supervisors.

Some may suggest that introducing students to opportunities abroad may contribute to the so-called ‘brain drain’. However, it has recently been suggested that this brain drain can be turned into a ‘brain gain’. According to Meyer and Brown,^3^ ‘There are two ways to implement the brain gain: either through the return of the expatriates to the country of origin (return option) or through their remote mobilisation and association to its development (diaspora option)’.

Postdoctoral experience abroad may introduce students to new ideas and techniques not available in South Africa. When these young researchers return, they bring some of these new techniques and ideas with them. Likewise, foreign students coming to South Africa for a postdoctoral position often bring new skills and ideas to South African laboratories.

Although the main aim of the workshop was to foster international collaboration, PhD students were also exposed to local opportunities. This was mainly through networking with local scientists at the workshop dinner. They also had the opportunity to listen to plenary talks by some of the most successful upcoming local researchers from the University of Cape Town (Prof Edward Sturrock, Dr Gasnat Shaboodien, Dr Liezl Zuhkl and Prof Sandrine Lecour), the University of Stellenbosch (Prof Faadiel Essop and Prof Hans Strijdom), and the University of KwaZulu-Natal (Prof Sajidah Khan).

Based on their presentations, there was a prize for the best South African and European PhD students. Ms Kathleen Reyskens from the University of Stellenbosch won the South African prize for her work on the effects of antiretroviral treatment on the heart. Ms Uma Mukherjee from the Hatter Institute at University College London won the European prize for her work on a molecule called DJ-1.

The student presentations were accompanied by plenary lectures from experts in cardiovascular research, and an ESC nucleus meeting of the working group in cellular biology of the heart, titled Frontiers in Cardioprotection, formed part of the programme. Finally, the meeting was concluded with a presentation by Prof Lionel Opie on how to become a successful researcher.

One of the highlights of the second joint meeting was that it extended past the UK borders, with the participation of other European PhD students and cardiovascular researchers. The next UK–SA (or UK–EU) workshop will be held in France in two years’ time.
